# Improving the Quality of Follow-Up Checklists in Rabak Teaching Hospital: A Two-Cycle Clinical Audit

**DOI:** 10.7759/cureus.82662

**Published:** 2025-04-20

**Authors:** Abubakr Muhammed, Mohammed AlSiddig Modawy Alkheder, Ibrahim Adil Hamadelniel Alhadi, Ayman Adil Eltayeb Abdelnour, Mustafa Sabir Abakar Awad, Asim Eisa, Rayan Elmutaz Mohamed Mahmoud, Seddig Alkazem Mohammed Alkheir, Mohammed Osman Ahmed Osman, Mohammed Eltayeb Elnour Elawad, Mie Sami Arbab Saeed, Abdelrahman Abbas Hassan Himed, Mustafa A AboAlella, Abdullah Mohamed, Tasneem Gadalla Elhag Adlan, Mogahid Hamdan Adam Ahmed, Thana Siddig Ahmed Yousif, Shahad Elhadi Ahmed Bagar, Areej Osman Adam Osman, Mohammed Abd Elgaioum Sailh Omer

**Affiliations:** 1 Surgery, University of Gezira, Madani, SDN; 2 General Surgery, Rabak Teaching Hospital, Rabak, SDN; 3 Internal Medicine, Rabak Teaching Hospital, Rabak, SDN; 4 Community Medicine, Al-Neelain University, Khartoum, SDN; 5 Oncology, Rabak Teaching Hospital, Rabak, SDN; 6 Radiology, Rabak Teaching Hospital, Rabak, SDN; 7 Pathology and Laboratory Medicine, Rabak Teaching Hospital, Rabak, SDN

**Keywords:** clinical audit, documentation quality, follow-up checklist, nice guidelines, patient safety

## Abstract

Background and aim: Follow-up checklists are essential tools in ensuring comprehensive patient care, promoting clear communication among healthcare professionals, and enhancing patient safety. This two-cycle clinical audit aimed to evaluate and improve the quality of follow-up checklists in Rabak Teaching Hospital by assessing adherence to standardized documentation practices based on the National Institute for Health and Care Excellence (NICE) guidelines.

Methods: A retrospective and prospective observational study was conducted over two cycles, with 50 follow-up notes evaluated in each cycle. The first cycle (baseline) was conducted between June 10, 2024, and July 12, 2024, and the second cycle (re-audit) was conducted from August 13, 2024, to August 24, 2024. Data were collected using a premade questionnaire based on NICE guidelines and analyzed using quantitative and qualitative methods.

Results: The second cycle revealed significant improvements in the completeness and accuracy of follow-up documentation compared to the first cycle. Key areas such as patient history, vital signs, physical examination, and management plans showed enhanced adherence to guidelines.

Conclusion: The findings demonstrate that structured follow-up checklists, combined with staff training and regular audits, can significantly improve documentation quality, thereby optimizing patient care. Continued re-auditing is recommended to sustain these improvements.

## Introduction

Clinical audits aim to enhance patient care by evaluating practices against standards and implementing changes, with follow-up checklists, such as those recommended by National Institute for Health and Care Excellence (NICE), playing a key role in standardizing documentation, improving communication, and ensuring patient safety [1-5].

Despite the proven benefits of follow-up checklists, many healthcare institutions still struggle with inconsistent documentation practices. A study by Pronovost et al. highlighted that the lack of standardized tools and inadequate staff training were major barriers to effective documentation [5,6]. These findings underscore the need for continuous quality improvement initiatives, such as clinical audits, to address these challenges, enhance patient care, and legal aspects.

This clinical audit builds on these global insights by evaluating the impact of standardized follow-up checklists and staff training on documentation quality in Rabak Teaching Hospital. The findings from this study contribute to the growing body of evidence supporting the use of structured documentation tools in healthcare settings.

## Materials and methods

Study design

This was a two-cycle retrospective and prospective observational study conducted at Rabak Teaching Hospital. The first cycle took place between June 10, 2024, and July 12, 2024, and the second cycle (re-audit) was conducted from August 13, 2024, to August 24, 2024.

Study area

The study was conducted at Rabak Teaching Hospital, a tertiary care facility in Sudan. The hospital serves a large patient population and provides a wide range of medical services, including internal medicine, surgery, and radiology.

Study population

The study population included all healthcare providers involved in writing follow-up notes in the internal medicine department. This included registrars, medical officers, and house officers. In each cycle, 50 follow-up notes were evaluated, totaling 100 notes across both cycles.

Sampling techniques

In each cycle, 50 follow-up notes were randomly selected from the hospital archives. The selection was based on the availability of notes written during the study period, ensuring a representative sample of documentation practices.

First cycle (baseline audit)

The first cycle of the audit was conducted between June 10, 2024, and July 12, 2024, and 50 follow-up notes from the internal medicine department were evaluated. The notes were completed by medical officers, registrars, and house officers. The results revealed significant gaps in documentation, particularly in the completeness of subjective and objective data. Only 22 (44%) notes included a detailed history of present illness (HPI), and 18 (36%) documented past medical history (PMH). Vital signs were recorded in 28 (56%) notes, and physical examination findings were documented in 24 (48%) cases.

Intervention

Between July 13, 2024, and August 12, 2024, several interventions were implemented to address the gaps identified in the first cycle. A standardized follow-up checklist based on NICE guidelines was introduced to ensure that all essential components of the SOAP framework were consistently included. Training sessions were conducted for healthcare providers, including registrars, medical officers, and house officers, to emphasize the importance of thorough documentation and the proper use of the new checklist. Additionally, continuous feedback was provided to staff on their documentation practices, with a focus on highlighting areas for improvement.

Second cycle (re-audit)

The second cycle was conducted on August 13, 2024, following the implementation of the interventions. A total of 50 follow-up notes were evaluated using the same criteria as the first cycle. Data were collected using a premade questionnaire based on NICE guidelines and analyzed using Google Forms (Menlo Park, CA: Google LLC) (appendix 1).

Data analysis

Quantitative and qualitative analyses were performed to assess the completeness and accuracy of the follow-up notes. The results were compared with those from the first audit cycle to evaluate improvements.

Ethical considerations

The audit was conducted in accordance with ethical guidelines for research and data collection. Patient confidentiality and privacy were maintained throughout the process. Approval was obtained from the hospital’s institutional review board (IRB) and ethics committee prior to commencing the audit.

## Results

The comparative analysis between the first and second audit cycles revealed significant improvements (p<0.05 for all criteria) across all documentation criteria. In the subjective components, chief complaint (CC) documentation showed a 30% increase from 30 (60%) to 45 (90%) (p<0.001), while complaint progression improved similarly from 25 (50%) to 40 (80%) (p<0.001). New complaint recording demonstrated substantial gains, rising from 20 (40%) to 35 (70%) (p=0.002). The history of present illness (HPI) showed the most notable improvement among subjective elements, increasing by 32% from 22 (44%) to 38 (76%) (p<0.001). Past medical history (PMH) documentation improved by 28% (18 {36%} to 32 {64%}; p=0.003), and current medications recording doubled from 15 (30%) to 30 (60%) (p=0.001).

Objective (O) documentation parameters exhibited consistent progress. Vital signs recording improved by 28% (28 {56%} to 42 {84%}; p<0.001), while physical examination documentation increased by 30% (24 {48%} to 39 {78%}; p<0.001). Laboratory results showed the most significant enhancement in this category, with a 32% improvement from 20 (40%) to 36 (72%) (p<0.001).

The assessment (A) and plan (P) components demonstrated particularly dramatic improvements. New diagnosis documentation increased by 30% (10 {20%} to 25 {50%}; p=0.001), while the recording of new management plans showed the most substantial overall improvement, rising by 32% (12 {24%} to 28 {56%}; p<0.001).

These results demonstrate comprehensive enhancements in clinical documentation practices, with improvement percentages ranging from 28% to 32% across all measured parameters (p<0.05 for all). The most substantial absolute improvements were observed in chief complaint (30 {60%} to 45 {90%}; p<0.001) and new management plans (12 {24%} to 28 {56%}; p<0.001). The consistent pattern of improvement suggests effective implementation of interventions, though some components (particularly in assessment and plan) remain below optimal levels, indicating areas for continued focus in future quality improvement initiatives (Table 1).

**Table 1:** Documentation quality improvements between first and second audit cycles. N (%): number (percentage) of cases where documentation was complete. Improvement (%): absolute percentage increase between cycles. P-value was calculated using McNemar’s test for paired proportions (comparing documentation completeness before and after intervention).

Criteria	First cycle, n (%)	Second cycle, n (%)	Improvement (%)	p-Value
Subjective (S)				
Chief complaint (CC)	30 (60%)	45 (90%)	30%	<0.001
Complaint progression	25 (50%)	40 (80%)	30%	<0.001
New complaint	20 (40%)	35 (70%)	30%	0.002
History of present illness (HPI)	22 (44%)	38 (76%)	32%	<0.001
Past medical history (PMH)	18 (36%)	32 (64%)	28%	0.003
Current medications	15 (30%)	30 (60%)	30%	0.001
Objective (O)				
Vital signs	28 (56%)	42 (84%)	28%	<0.001
Physical examination	24 (48%)	39 (78%)	30%	<0.001
Laboratory results	20 (40%)	36 (72%)	32%	<0.001
Assessment (A)				
New diagnosis	10 (20%)	25 (50%)	30%	0.001
Plan (P)				
New plan of management	12 (24%)	28 (56%)	32%	<0.001

These findings demonstrate significantly enhanced compliance with NICE guidelines and improved clinical documentation (p<0.01 for all comparisons). The results suggest better interdisciplinary communication and strengthened patient safety measures, while highlighting the value of continued training and periodic re-audits to maintain these standards.

## Discussion

The findings of this two-cycle clinical audit underscore the significant impact of structured follow-up checklists and staff training on improving the quality of patient documentation in Rabak Teaching Hospital. The marked improvements observed in the second cycle, particularly in the completeness and accuracy of subjective, objective, assessment, plan (SOAP) documentation, highlight the effectiveness of the interventions implemented after the first cycle. These interventions included the introduction of standardized follow-up checklists based on NICE guidelines, targeted staff training sessions, and continuous feedback mechanisms. The results align with global evidence demonstrating that structured documentation tools and regular audits can enhance patient care by reducing errors, improving communication, and ensuring adherence to clinical guidelines [1,2].

The improvements in documentation quality observed in this study are consistent with findings from other global studies. For instance, Johnston et al. emphasized the role of clinical audits in identifying gaps in healthcare delivery and implementing changes to improve patient outcomes [2]. Similarly, a study by Hales and Pronovost found that the use of checklists in healthcare settings significantly improved the accuracy of patient records and reduced errors in clinical practice [3,7]. These studies, like ours, highlight the importance of continuous quality improvement initiatives in healthcare. Furthermore, the study by Grol and Grimshaw demonstrated that the implementation of evidence-based guidelines, such as those provided by NICE, can lead to significant improvements in clinical practice [5]. The findings of this study support this, as adherence to NICE guidelines in the second cycle resulted in better documentation of patient history, vital signs, and management plans.

The use of checklists in healthcare settings has been widely studied and proven effective in improving patient safety and outcomes. Pronovost et al. conducted a study on the use of checklists in intensive care units and found that they significantly reduced errors and improved patient safety [5]. Similarly, a study by Treadwell et al. demonstrated that the use of checklists in surgical settings reduced complications and improved patient outcomes [7,8]. These findings are consistent with our results, which showed that standardized follow-up checklists improved the documentation of patient care, potentially reducing the risk of complications. Additionally, a study by Berenholtz et al. highlighted the importance of staff engagement and training in the successful implementation of clinical audits [8,9]. Our study also emphasized the role of staff training and engagement in achieving improvements in documentation quality.

Limitations

Despite the significant improvements observed in this audit, some limitations must be acknowledged. This study was conducted at the department of internal medicine in a single hospital, which may limit the generalizability of the findings to other healthcare settings. Additionally, the short duration of the audit period may not fully capture long-term trends in documentation practices. Future studies should consider extending the audit period and including multiple healthcare facilities to provide a more comprehensive assessment of the impact of standardized checklists and staff training on documentation quality.

## Conclusions

This two-cycle clinical audit demonstrates that structured follow-up checklists, combined with staff training and regular audits, significantly improve the completeness and accuracy of patient documentation at Rabak Teaching Hospital. Enhanced documentation is a critical first step toward optimizing patient care by promoting clarity, continuity, and accountability. This study did not directly assess patient outcomes or safety metrics. Future work should evaluate how these documentation improvements translate into tangible clinical benefits, such as reduced errors or improved treatment efficacy. Sustaining these gains will require ongoing re-auditing, staff reinforcement, and integration of feedback mechanisms.

## Figures and Tables

**Figure 1:**
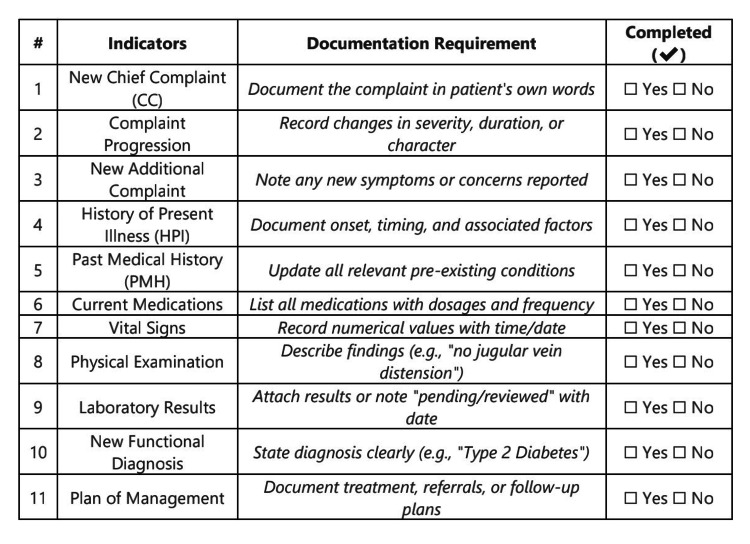
Follow-up checklist questionnaire.

## References

[1] (2019). Surgical site infections: prevention and treatment. https://www.nice.org.uk/guidance/ng125.

[2] Johnston G, Crombie IK, Davies HT, Alder EM, Millard A (2000). Reviewing audit: barriers and facilitating factors for effective clinical audit. Qual Health Care.

[3] Hales BM, Pronovost PJ (2006). The checklist - a tool for error management and performance improvement. J Crit Care.

[4] Grol R, Grimshaw J (2003). From best evidence to best practice: effective implementation of change in patients' care. Lancet.

[5] Pronovost P, Needham D, Berenholtz S (2006). An intervention to decrease catheter-related bloodstream infections in the ICU. N Engl J Med.

[6] Haynes AB, Weiser TG, Berry WR (2009). A surgical safety checklist to reduce morbidity and mortality in a global population. N Engl J Med.

[7] Treadwell JR, Lucas S, Tsou AY (2014). Surgical checklists: a systematic review of impacts and implementation. BMJ Qual Saf.

[8] Berenholtz SM, Pronovost PJ, Lipsett PA (2004). Eliminating catheter-related bloodstream infections in the intensive care unit. Crit Care Med.

[9] Winters BD, Gurses AP, Lehmann H, Sexton JB, Rampersad CJ, Pronovost PJ (2009). Clinical review: checklists - translating evidence into practice. Crit Care.

